# High flux circularly polarized gamma beam factory: coupling a Fabry-Perot optical cavity with an electron storage ring

**DOI:** 10.1038/srep36569

**Published:** 2016-11-18

**Authors:** I. Chaikovska, K. Cassou, R. Chiche, R. Cizeron, P. Cornebise, N. Delerue, D. Jehanno, F. Labaye, R. Marie, A. Martens, Y. Peinaud, V. Soskov, A. Variola, F. Zomer, E. Cormier, J. Lhermite, V. Dolique, R. Flaminio, C. Michel, L. Pinard, B. Sassolas, T. Akagi, S. Araki, Y. Honda, T. Omori, N. Terunuma, J. Urakawa, S. Miyoshi, T. Takahashi, H. Yoshitama

**Affiliations:** 1 Laboratoire de l’Accélérateur Linéaire (LAL), Univ. Paris-Sud, CNRS/IN2P3, Université Paris-Saclay, Orsay, 91898, France; 2 Centre Lasers Intenses et Applications (CELIA), CNRS/CEA, Université de Bordeaux, Talence, 33405, France; 3 Laboratoire des Matériaux Avancés (LMA), CNRS/IN2P3, Villeurbanne, 69622, France; 4 High Energy Accelerator Research Organization (KEK), Tsukuba, 305-0801, Japan; 5 Hiroshima University, Graduate School of Science, Higashi-Hiroshima, 739-8511, Japan

## Abstract

We report and discuss high-flux generation of circularly polarized *γ*-rays by means of Compton scattering. The *γ*-ray beam results from the collision of an external-cavity-enhanced infrared laser beam and a low emittance relativistic electron beam. By operating a non-planar bow-tie high-finesse optical Fabry-Perot cavity coupled to a storage ring, we have recorded a flux of up to (3.5 ± 0.3) × 10^8^ photons per second with a mean measured energy of 24 MeV. The *γ*-ray flux has been sustained for several hours. In particular, we were able to measure a record value of up to 400 *γ*-rays per collision in a full bandwidth. Moreover, the impact of Compton scattering on the electron beam dynamics could be observed resulting in a reduction of the electron beam lifetime correlated to the laser power stored in the Fabry-Perot cavity. We demonstrate that the electron beam lifetime provides an independent and consistent determination of the *γ*-ray flux. Furthermore, a reduction of the *γ*-ray flux due to intrabeam scattering has clearly been identified. These results, obtained on an accelerator test facility, warrant potential scaling and revealed both expected and yet unobserved effects. They set the baseline for further scaling of the future Compton sources under development around the world.

High-intensity X- and *γ*-ray beams are extremely useful in fundamental and applied physics as well as in the fields of medicine, material studies, cultural heritage preservation, etc.^1^,^2^,^3^,^4^,^5^. In this context, accelerator driven Compton *γ*-ray sources, where the collisions between laser pulses and relativistic electron bunches result in the production of high-energy photons, have a renewed interest owing to the recent developments in laser and accelerator technologies. Indeed, several Compton facilities are under construction or operation all around the world^6^,^7^,^8^,^9^,^10^,^11^. For the *γ*-ray facilities, especially for the facilities providing the *γ*-rays with an energy cutoff above 1 MeV, the Compton sources represent almost a unique opportunity. In fact, thanks to the large photon energy boost they can provide high-energy *γ*-rays with a relatively low energy electron beam. This allows reaching the 1–20 MeV photon energy range with the GeV classes facilities providing the possibility to build the infrastructures for the applications in the nuclear and fundamental physics field. In the same context, a unique feature is given by the Compton effect energy-emission angle biunivocal relationship. New experiments and techniques like, for examples, Nuclear Resonance Fluorescence (NRF) or axions particle-like search, need monochromatic high-energy photon beams in the level of 0.1%. In this energy range there are no monochromators available and the only way to produce these unique performances is to select, with a precise collimation system, the central part of the Compton scattering emission cone. On the other hand, the future *γ*–*γ* collider^12^,^13^,^14^ has foreseen to generate the *γ*-rays at higher energy range (above 100 GeV) by Compton scattering. The interest of such facilities has been renewed in the context of the recent indications of a new high mass diphoton resonance observed at the Large Hadron Collider (LHC)^15^,^16^.

Unfortunately, the scattering Compton cross-section is relatively low and thus only a small fraction of the electrons and photons interacts leading to a limited *γ*-ray production. In the quest for high flux *γ*-ray generation, two ways can be considered. Thus, increasing the Compton flux would require a higher photon flux and/or a higher electron current. However, this approach is limited by the technological constraints and after a certain regime by the intrinsic limitations owing to non-linear Compton scattering. Since most of the X- and *γ*-ray facilities usually seek for the high average flux, the Compton flux, in this case, can be increased by employing a higher repetition rate of the collisions implying the moderate photon and electron intensities. Moreover, the high stability of the scattered flux at such facilities is of great importance. Therefore, an alternative approach to enhance the scattered photon production would consist in recycling the electrons (in a storage ring) and/or the photons (in a high finesse Fabry-Perot Cavity for instance).

Based on our previous experience^17^ we have built a non-planar bow-tie Fabry-Perot Cavity (FPC)^18^ to store an infrared laser beam with up to 50 kW of average power consisting of picosecond pulses circulating at a repetition rate of 178.5 MHz making its design and properties different from that of higher frequencies and higher finesse cavities^19^. Such design of the FPC has the advantage of being more stable when providing a small beam waist ensuring circularly polarized fundamental eigenmodes and has been adopted given the mechanical constraints of the installation at the accelerator facility. The polarized *γ*-rays have been produced by installing the optical cavity and its laser system at the Accelerator Test Facility (ATF)^20^ at KEK in Tsukuba, Japan. The ATF Damping Ring (DR) provides electron bunches with an energy of 1.28 GeV and the experiment consisted in properly setting the conditions for optimal interaction between the laser pulses and the electron bunches with perfect timing as collisions occur at 1.08 MHz. Note here that the ring can also be injected with more complex electron patterns (multi-bunch operation mode) thus increasing further the final *γ*-ray flux. Our system is sufficiently stable to sustain a high-flux *γ*-ray production for several hours.

It is worth mentioning that the high power optical recycling technique in a FPC, as presented here, has also applications in other contexts. For instance, one could replace the electron beam colliding with the cavity optical beam in the focal plane by an atomic or molecular gas jet. A highly non-linear interaction will produce a XUV beam with a high degree of both spatial and temporal coherence at a very high repetition rate^21^, which is commonly used for metrology, molecular spectroscopy, electron dynamics studies with attosecond time resolution. For the same reasons as for the Compton scattering applications, the interaction inside the optical cavity is prone to high fluxes production. Additionally, the FPC acts as an optical power storage able to handle average power in the range 10 kW–1 MW. Since we operate the system in a pulsed mode, the passive gain observed on the average power applies on the pulse energy as well^22^. It, therefore, would be interesting to extract the circulating high energy pulse when necessary. It is the purpose of the ongoing research topic of high finesse passive cavity damping, where the advances presented will certainly benefit^23^.

Thanks to the cavity design (allowing tight focussing) and finesse (high stored power) together with the very low emittance of the DR (small electron beam size) we were able to record an outstanding value of up to 400 *γ*-rays per collision with the energy cutoff of 30 MeV in a full bandwidth. Since a main purpose of this experiment being the demonstration of the performances of a FPC working in the accelerator environment to produce high *γ*-ray flux, no optimization in terms of the generated *γ*-ray bandwidth was possible. However, it is known that low emittance and energy spread in a ring are mandatory to reach a very low bandwidth of the emission spectrum if the relatively large collimator aperture and small beam dimensions are employed to maximize the *γ*-ray production^7^. In this context, the experimental results exposed in this Report showing the preservation of the low emittance in the ATF DR in presence of Compton scattering and given no electron energy spread increase measured during the experiment imply a potential of reaching a low *γ*-ray bandwidth.

Although, as mentioned earlier the Compton scattering cross-section is very low, we demonstrate and discuss here yet unobserved large effect on electron beam dynamics that is due to the *γ*-ray production efficiency we finally reached thanks to both beam recycling. Based on the present results, implications and scaling for future X- and *γ*-ray Compton scattering facilities are discussed in following sections.

## Results

**Figure 1:**
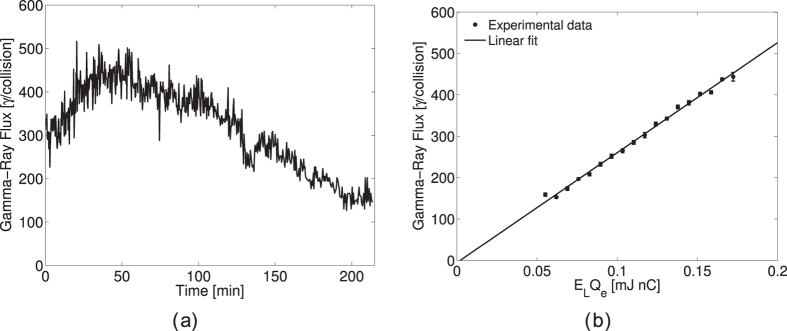
(**a**) Number of *γ*-rays produced versus time in the single-bunch operation mode. (**b**) Number of *γ*-rays produced per interaction as a function of the laser pulse energy *E*_*L*_ and the electron bunch charge *Q*_*e*_ product. The data points are fitted with a first order polynomial. The error bars represent the standard error on the mean value.

Electron-photon collision based experiments in accelerator facilities usually require very stable beams over long periods of time of typically several hours. In case, where the laser beam power is boosted in an external FPC, the constraints are even more severe. In fact, besides the beam stability, one has to keep the laser oscillator locked on the FPC with sufficient coupling. Thanks to the high stability of the laser, the amplifier and the home-made feedback system, we could maintain a high rate of *γ*-ray production during several hours on a daily base. The *γ*-ray production displayed on Fig. 1a was recorded during a 4 hours run with outstanding peak values in excess of 400 *γ*-rays per collision. It should be pointed out that due to the accelerator operation constraints and given the location of the FPC installation in the DR, the parameters of the electron beam in the interaction point have not been optimized during the Compton scattering experimental campaign. The fluctuations and variations in time are directly related to fluctuations of the electron current and the average power stored in the FPC. In order to decouple effects, the accelerator is operated in single-bunch mode for which the Compton collisions occur every other DR round trip at an effective rate of 1.08 MHz. The rate is predominantly influenced by the laser power in the FPC or more precisely the circulating pulse energy *E*_*L*_ and the charge *Q*_*e*_ of the electron beam that is measured with a DC current transformer (DCCT). In Fig. 1b we plot the number of *γ*-rays produced per collision as a function of the product *E*_*L*_*Q*_*e*_ (i.e. the number of laser photons times the number of electrons) over a large range (in practice by changing the laser power as the electron current in the ring is rather fixed). As expected from the theory, the curve follows a linear behaviour with no trace of saturation revealing a non-depletion regime. The curve slope indicates that 2660 ± 190 *γ*/(mJ ⋅ nC) are produced at each interaction. The uncertainty here is related to the calorimeter calibration evaluated at 68% Confidence Level (CL), statistical uncertainty was in this case 25 *γ*/(mJ ⋅ nC).

This number can be scaled to any facility that uses a similar scheme to produce X- or *γ*-rays through Compton scattering. In our case, with an electron bunch charge of 1 nC, a typical average laser beam power of 35 kW, a collision frequency of 1.08 MHz and a duty cycle of the ATF DR of 0.62, it corresponds to the generation of (3.5 ± 0.3) × 10^8^ photons per second, where the uncertainty includes both statistical and systematic uncertainties. This performance is competitive with current *γ*-ray facilities. More importantly, we have demonstrated the production of approximately 400 photons per interaction which is, to the best of our knowledge, a record for existing Compton facilities at this energy range.

According to the kinematics of Compton scattering the scattered photons possess the polarization of the laser photons^24^,^25^. In such a way, by using linear or circularly polarized laser beam two states of the scattered photon polarization can be generated. Although measurement and description of the *γ*-ray polarization are out of the scope of this experiment, the circularly polarized laser beam together with the FPC design implies, in a restricted area of the emitted radiation, production of the circularly polarized *γ*-rays^26^,^27^. In this case, the final achievable polarization scales as a function of the *γ*-ray collimator aperture and depends on the quality of the electron and laser beams.

## Discussion

**Figure 2:**
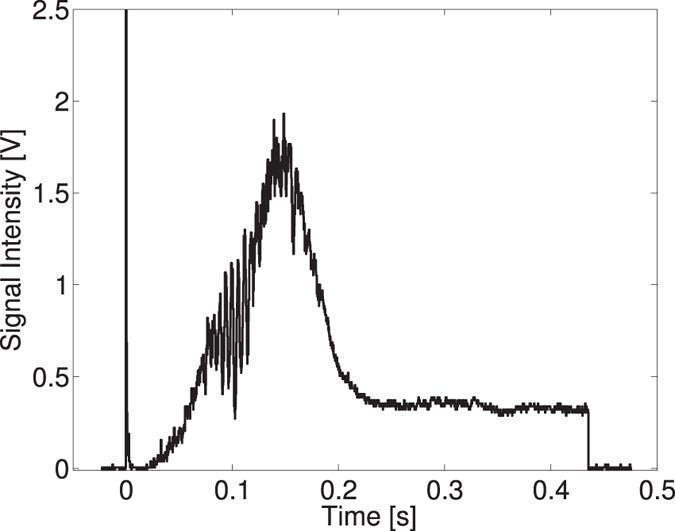
Raw signal showing the time evolution of the *γ*-ray flux over 450 ms.

The luminosity of the Compton collisions strongly depends on the size of the electron beam^28^ and so reflects the evolution of the electron beam emittance in the DR during which the electron beam eventually reaches an equilibrium determined by a balance between the radiation damping and the quantum excitation^29^. However, the collective effects such as intrabeam scattering, coherent synchrotron radiation, wakefields, etc. can also have a noticeable effect depending on the beam parameters and the accelerator design. They can be responsible for additional energy spread, bunch lengthening and beam instabilities. In the ATF DR, at high beam intensity the strong effects of intrabeam scattering are known to occur^30^ due to an extremely low value of the transverse emittance. When a high flux of photons is produced by Compton scattering, the effect of intrabeam scattering is instantaneously reported in the time evolution of the recorded *γ*-ray flux. Therefore, Compton scattering provides in this context an ultrafast diagnostic tool to analyse the electron beam dynamics. This effect is shown in Fig. 2, where a lower injection frequency of the ATF DR of 1.56 Hz is used, thus allowing to store the electron beam for 450 ms (9.7 × 10^5^ round trips). The measured signal intensity as a function of time is illustrated in Fig. 2. The effect of intrabeam scattering in the ATF DR manifests itself as a modification of the transverse shape (together with the momentum spread) of the beam and thus, in a modification of the luminosity that in turn depends on the size of the electron beam. The high intensity peak at the beginning of the waveform is due to a large background generated by the injection of the electrons in the DR. The origin of the *γ*-ray signal oscillations seen at the beginning of the waveform for the moment stays unclear. These oscillations often occurred during the runs and can be caused by the instabilities or modulation of particle density induced by the beam instabilities. In general, the temporal shape of the gamma ray pulses could be explained as follows. When the electron beam is injected it has a large emittance and therefore the *γ*-ray flux produced is small. As the beam sizes reduce due to radiation damping the emission rate increases (up to 150 ms after injection in the DR). However, when the beam emittance becomes smaller than a certain amount, the intrabeam scattering is becoming important leading to an increase in beam emittance and is inducing a decrease of the photon flux (between 150 and 230 ms after injection in the DR) until a new equilibrium state is established (from 230 ms after injection in the DR and up to extraction of the beam). Based on the present analysis, one can take advantage of intrabeam scattering to further optimize the *γ*-ray production since the transient emission (below 230 ms) is several times higher than the steady state emission (after 230 ms). There exists an optimal time for which the electron bunch should be extracted and a new bunch injected.

**Figure 3:**
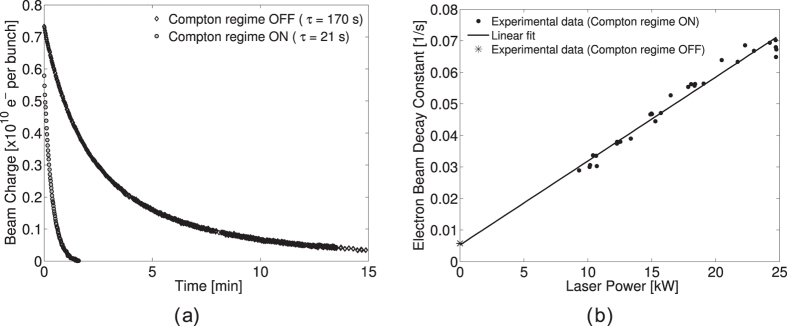
(**a**) Electron beam charge as a function of time in absence and presence of Compton scattering. A reduction by a factor 8 of the beam lifetime is observed due to the presence of Compton scattering. The laser power stored in the Fabry-Perot cavity during this measurement is 15 kW. (**b**) Decay constant as a function of the laser power stored in the Fabry-Perot cavity. The data points are fitted with a first order polynomial. The data points taken in absence of Compton scattering are excluded from the fitting procedure.

During the Compton process, electrons are scattered from their initial trajectory with an energy loss matching the energy of the produced photons. Since the momentum acceptance of the ATF DR is (*δ*p/*p*)_max_ = 0.012, corresponding to a RF bucket height of approximately 15 MeV, a low momentum transfer between the electron and the photon will retain the electrons within the separatrix whereas larger perturbations, such as Compton scattering with high momentum transfer, typically *δp* > 15 MeV, will eject the concerned electrons out of the RF bucket resulting in beam loss. Since photons with energies higher than 15 MeV are often produced by Compton scattering, this effect should be observed on the electron beam lifetime, in particular in our configuration, where the scattering rate is significant. In absence of Compton scattering, the ATF DR electron beam lifetime is mainly determined by elastic beam-gas scattering and Touschek effect^31^. In an experiment, where the ring was not periodically reinjected, the electron beam charge has been measured with a DCCT as a function of time in absence and presence of Compton scattering (see Fig. 3a). The shortening by approximately a factor 8 of the electron beam lifetime due to the presence of Compton scattering is clearly visible (the electron beam lifetime is reduced from 170 s down to 21 s with collisions). The beam lifetime is defined here as the time interval after which the beam charge has reached 1/*e* of its initial value. As expected, the inverse of the electron beam lifetime, so-called decay constant, depends linearly on the laser power stored in the FPC. This dependence is shown in Fig. 3b. The data in presence of the Compton collisions are fitted with a linear curve that predicts fairly well the data points taken in absence of collisions. Indeed, the linear fit gives an intercept of 0.0053 ± 0.0031 s^−1^ at 95% CL while the inverse electron beam lifetime (without Compton collisions) is measured to be 0.0058 s^−1^ with negligible uncertainty compared to the fit. The slope of the curve is extracted to be 0.0027 ± 0.0002 s^−1^ kW^−1^ at 95% CL. Such a measurement is of prime importance to discuss power scaling of *γ*-ray production in this context. In fact, we just demonstrated that in a high-flux configuration the Compton interaction has a significant impact on the beam dynamics. Although operating a FPC in an accelerator environment at a 50 kW level is not trivial, state of the art power storage of picosecond pulses in the FPC have reach 600 kW^22^. The obtained results can predict whether the electron beam could survive such an average laser power. For a targeted optical power of 1 MW, Fig. 3b indicates a decay constant of 2.7 s^−1^ (all other factors being equal) implying the need to reinject electron bunches at more than 2.7 Hz which is very close to the standard injection frequency of 3.13 Hz at the ATF.

Accidentally the beam lifetime analysis can also provide an accurate determination of the *γ*-ray flux that is independent on the calorimeter calibration.

Assuming a constant beam lifetime that exhibits a weak dependence on the beam charge, the total decay constant in presence of Compton scattering can be given as a sum of the initial decay constant and the one caused by Compton scattering. Since the latter is mainly defined by the number of the scattered *γ*-rays and a probability to scatter the *γ*-ray outside the DR momentum acceptance, one can estimate the *γ*-ray rate from the beam lifetime measurements. In such a way, taking the decay constant at different values of the laser power, we found that linear parametrisation gives the *γ*-ray rate of 2010 ± 100 *γ*/(mJ ⋅ nC) at 95% CL which is in agreement within 25% with the direct measurement. This discrepancy at the level of three standard deviations can be explained either by an underestimated systematic error in the calibration procedure or by a potential dependence of the electron beam lifetime on its charge owing to the processes involved in the determination of the beam lifetime.

Since the ATF architecture is fixed, scaling will be mainly driven by the optical power scaling. With a stored laser power of 500 kW and three macropulses having 10 electron bunches of 1 nC each (maximum configuration of the ATF DR) the total *γ*-ray flux would reach 1.5 × 10^11^ photons per second on a test facility.

At present, two main facilities exhibit large fluxes of *γ*-rays allowing to perform nuclear physics experiments. The HI*γ*S facility located at Duke University in Durham, NC, USA is the most intense source. It delivers up to 10^10^ photons per second in the range of 1 MeV to 100 MeV^32^. Photons are produced in bunches at a rate of 5.6 MHz approximately. Note that they have reported a flux of up to 5 × 10^8^ that is about 100 *γ*-rays per collision with the Compton energy cutoff of 30 MeV^33^. However, the interaction laser consists in a Free Electron Laser whose complexity and cost are in no way comparable to a fiber laser coupled to a FPC. The technique described in this Report is intended to be used in an upgrade proposal, HI*γ*S2 facility. In this case, the *γ*-rays may be produced with a rate of 10^11^–10^12^ photons per second by using a fiber laser that seeds a FPC to enhance the laser power up to 20 kW. The pulse rate of the *γ*-ray beam will be 89.3 MHz^34^.

Another major facility, newSUBARU located at Hyogo University in Japan produces a high flux of *γ*-rays of up to 10^6^ photons per second with a 4 W Q-switched Nd laser^35^. The production rate could be largely increased if one imagines an upgrade of this machine with the technique presented in this Report. Indeed, assuming the use of the FPC at 250 MHz and the stored power of 30 kW, the flux of the *γ*-rays could be increased by a factor of 7000. This number only takes into account the gain in flux related to the laser power and the repetition rate of the collisions.

A compact Compton X-ray facility called ThomX is currently under construction at LAL in Orsay by some of us^8^. It will use the FPC with an optical length of about 9 m. With 30 kW of stored laser power, the electron bunches of 1 nC and taking into account the collision rate of 16.67 MHz, by using the experimentally obtained value of the *γ*-ray flux, one obtains about 4 × 10^10^ photons per second and 8 × 10^11^ photons per second assuming 600 kW stored power, which is the state of the art in terms of stored power in the FPC in the pulsed regime^22^. These numbers are not taking into account the improvements in the laser coupling to the FPC and a better matching of the beam sizes owing to a more optimized design in the case of ThomX compared to the experiment exposed in this Report.

Finally, another approach is employed for the Compton *γ*-ray facility ELI-NP-GBS in construction in Magurele, Romania. It will deliver photons with energies ranging from 0.2 MeV to 20 MeV with an excellent spectral density of photons reaching 5000 ph/(s ⋅ eV) at 2 MeV and 10 MeV and the photon bandwidth ≤0.5% needed for the NRF experiments^7^. It was chosen to implement a linac with an optical circulator^36^ instead of an electron ring and a FPC. In this case, using a FPC in burst mode is also a possible solution. The obtained results of the high finesse pulsed FPC presented here along with the recent developments in burst mode pulsed FPC at low power^37^ may be combined for future compact and highly performant photon sources based on the Compton scattering process.

Based on the performances achieved in this experimental campaign, the *γ*-ray flux has been scaled to other facilities, where the FPC could be or is being implemented in the pulsed regime. The obtained results demonstrate the great advantage of using such a scheme despite the difficulties related to the high finesse and the operation of the optical cavity in pulsed regime.

## Methods

### Electron damping ring

The ATF comprises a S-band linac, a 138 m circumference DR and an extraction line. The ATF DR^38^ is a low emittance storage ring with a revolution frequency of 2.16 MHz, where 1.28 GeV electrons are stored. The length of the FPC has been chosen to synchronize the optical pulse train with the electron bunches of the ATF DR. It allows the production of *γ*-rays in the multi-bunch operation mode of the accelerator. Up to three macropulses can be injected into the DR, with up to ten bunches per train at 5.6 ns bunch spacing. Although, the electrons are typically injected at 3.13 Hz from the linac in the DR and stored for about 250 ms before being extracted, other injection patterns can be employed. During the standard operations of the ATF a single bunch is injected and stored in the DR. The beam intensity in the single-bunch operation can reach 1.6 nC (3.46 mA). After about 200 ms the electron beam is fully damped. The FPC is installed in one of the straight sections of the DR. The produced *γ*-rays propagate along with the electron beam before being extracted through a window in the vacuum chamber. More details on the experiment are given elsewhere^39^.

### Ultralow-noise high-power laser system

**Figure 4:**
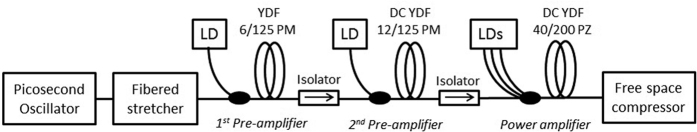
Schematic diagram of the high power picosecond pulsed laser, where LD and DC YDF denote a laser diode and a double clad Ytterbium doped fiber, respectively.

The laser system is based on an all-fibered Master Oscillator Power Amplifier system using Chirped Pulse Amplification as depicted in Fig. 4. The seed is a commercial frequency comb oscillator delivering approximately 20 mW of optical average power. The mode-lock operation is obtained by non-linear polarization evolution in an all-normal dispersion regime. It delivers naturally chirped pulses with approximately 2 ps duration at a repetition rate of 178.5 MHz.

**Figure 5:**
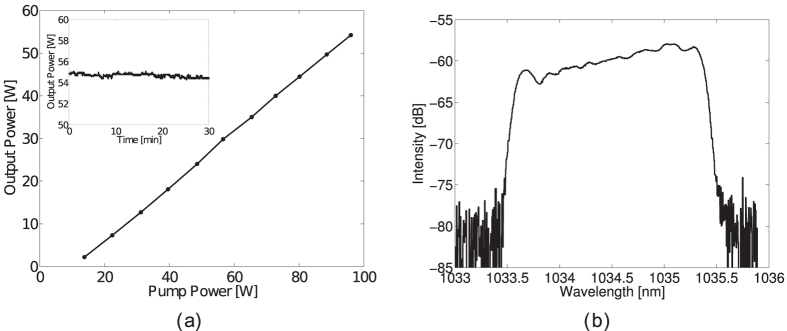
(**a**) Output power versus the pump power of the amplified laser beam. (**b**) Emission spectrum of the amplified laser beam. The square shape of the emission spectrum is due to the transmission bandwidth of the stretcher and compressor couple.

The pulses are stretched up to 220 ps through a linearly Chirped Fiber Bragg Grating (CFBG) specifically designed for our set-up to prevent deleterious nonlinear effects such as undesired spectral broadening by self-phase modulation (SPM) and most important AM-FM couplings. These stretched pulses are amplified in three successive Ytterbium-doped amplification stages pumped at 976 nm. They are isolated with fiber pigtailed isolators to avoid reverse propagation of parasitic signal. The first stage is a core pumped polarization maintaining (PM) Ytterbium-doped fiber (YDF) amplifier designed with a standard 6/125 *μ*m fiber and delivering 100 mW of average power. The second one consists of a double clad 12/125 *μ*m PM YDF. This stage is cladding pumped through a 2 + 1 to 1 combiner and delivers an average power around 1 W. The power amplification stage includes a NKT photonics DC-40/200-PZ-Yb-doped fiber. It is seeded and pumped through a 6 + 1 to 1 combiner spliced to the active fiber. The coupling of the signal to the core and of the pump to the clad is optimized thanks to a mode field adapter specially designed for this amplification stage. The output power reaches 54 W for a pump power of 96 W (see Fig. 5a). The slope efficiency of the amplifier reaches 64% once the losses are compensated. The measured output power exhibits variations below 1% peak-to-peak over 30 minutes, (see insert of Fig. 5a). The laser-beam is compressed with a Chirped Volume Bragg Grating (CVBG) designed to obtain a pulse duration of about 10 ps which maximizes the temporal overlap of the laser and electron beams and in turn the luminosity of the interaction. The experimental pulse duration corresponding to a FWHM of 11.4 ps, is measured by means of an auto-correlator. The corresponding emission spectrum is depicted in Fig. 5b.

### High finesse Fabry-Perot optical cavity

The FPC is formed by two concave mirrors with a radius of curvature of 500 mm and two flat mirrors. A detailed description is given in ref. 40. Taking into account that a width of the resonance peak is inversely proportional to the cavity finesse, with a finesse of approximately 30000 and an optical path length of 1679.52 mm, the precision required on this length is approximately 35 pm. Therefore, in order to maintain the cavity in resonance, the optical length of the cavity and the laser repetition frequency must match with a very high precision. This is achieved by acting on a piezo-electric transducer (PZT) that modulates the length of the cavity of the laser oscillator by means of an electronic feedback system based on the Pound-Drever-Hall technique^41^. As the crossing of the electron and laser beams must be synchronous, the optical length of the FPC is also adjusted to the reference clock of the accelerator. This is performed using a Phase Lock Loop (PLL) that controls a PZT located behind one of the mirrors of the FPC to slightly adjust the length of the optical path inside the cavity^40^.

**Figure 6:**
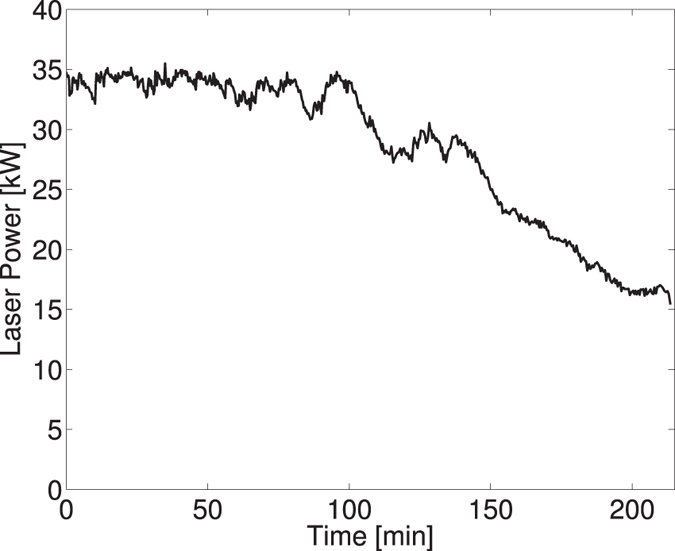
Laser power stored in the Fabry-Perot cavity measured during one of the data taking.

Intra-cavity power is determined by measuring the beam power leaking out of any of the cavity mirrors for which the transmission has been characterized to very high accuracy. The actual stored power obviously depends on the cavity finesse and the incident laser power however is very sensitive to the mode matching between the laser beam and the eigen-mode of the cavity as well as the frequency comb matching of both the oscillator and the passive cavity as optimal coupling is obtained at resonance. Figure 6 shows a typical run, where the cavity average power was maintained above 30 kW for about 2 hours. The slow decrease occurring after two hours is due to an excessive low frequency drift of the carrier to envelope phase of the laser the feedback system could not compensate. In fact, the carrier to envelope phase slow drift of the laser, leading to a cavity gain decrease^42^, is compensated by acting on the isochronic wedges^43^ inserted in the laser oscillator. At some point this compensation is not possible and the stored laser power decreases. Due to the change in the thermal load, the distance between the concave mirrors of the FPC must be adjusted until the end of the full range of the actuators is reached.

The maximum power stored in the cavity during the runs is approximately 35 kW. This value is limited by the thermoelastic deformation of the FPC mirrors^22^. Although of extreme reflectivity (<1 ppm of absorption) the intra-cavity beam average power locally heats the mirrors inducing a deformation partially changing their radii of curvature and thus the spatial eigen-mode. It in turn prevents an efficient coupling with the incident laser beam. In order to compensate for the thermal distortion, we moved the two concave mirrors apart. The resulting cavity eigen-mode is scaled up leading to a larger spot size on the mirrors. Although the same amount of heat is deposited, the gradient is reduced leading to a weaker thermal lensing. After adjusting the laser beam to match the new cavity eigen-mode, the power stored in the cavity reached 46 kW in a steady state regime.

### Gamma-ray detection and alignment procedure

**Figure 7:**
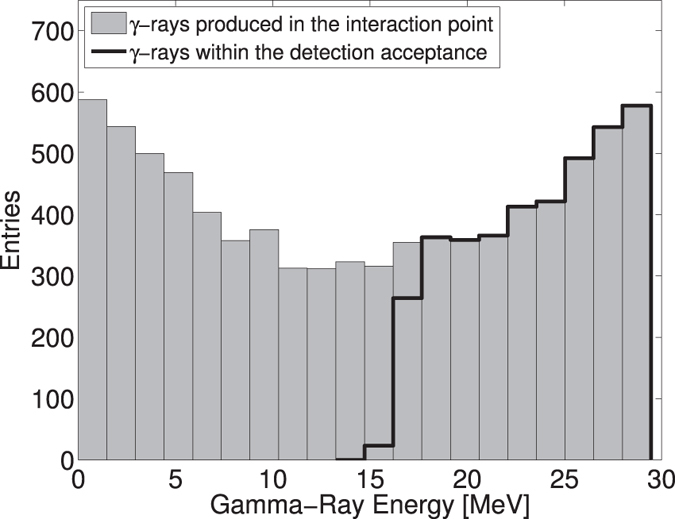
Computed energy spectrum of the *γ*-rays produced by Compton scattering of a 1034 nm laser beam off a 1.28 GeV electron beam. Only the *γ*-rays with energies above 15 MeV are detected resulting in the average energy of the measured *γ*-rays being 24 MeV.

The *γ*-rays produced during the collisions are recorded by a detector installed about 20 meters downstream the interaction point. It is a scintillation detector coupled to a photomultiplier tube. Figure 7 shows the simulated energy spectrum of the *γ*-rays taking into account the experimental parameters^44^. During the experiment, the scattered *γ*-rays pass through several collimators before being detected. This results in the energy selection owing to the energy-angle correlation in Compton scattering. Description of the detector together with the details on the expected *γ*-ray spectrum and experimental parameters are summarized in ref. 39. The *γ*-ray detector is calibrated on the steady state cosmic rays flux impinging on the calorimeter taking into account the detailed geometry of the calorimeter. The procedure is described in detail in ref. 45. The detector signal is recorded with an oscilloscope after a trigger signal related to the injection of the electrons in the DR is found. The total energy of the *γ*-ray beam is thus measured during one DR storage period.

**Figure 8:**
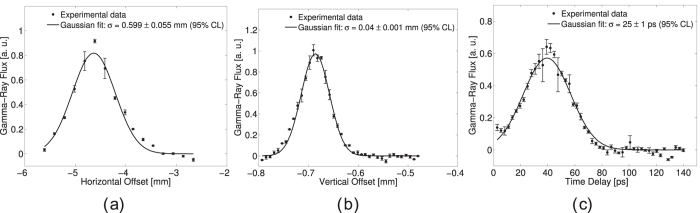
(**a**) Measured *γ*-ray flux as a function of the horizontal position of the Fabry-Perot cavity relative to the electron beam. (**b**) Measured *γ*-ray flux as a function of the vertical position of the Fabry-Perot cavity relative to the electron beam. (**c**) Measured *γ*-ray flux as a function of the phase shift between the reference clock of the accelerator and the laser oscillator responsible for the synchronization of the passage of the laser and electron beams at the interaction point. The error bars represent the standard error on the mean value.

The optical cavity and the laser system are sitting on a pile of 2 optical tables separated by vibration isolators. The 3 tons vessel is equipped with actuators allowing to move the entire bench with micron precision in the three dimensions. Therefore, the FPC waist can be finely positioned to maximize the overlap with the unmovable electron beam. This feature is used at the beginning of the campaign to align the FPC with the electron beam and several times after that to check the stability of the alignment. The optimum alignment is found when the *γ*-ray flux is maximized during scans in three spatial dimensions. A scan of the phase difference between the reference clock of the accelerator and the laser oscillator is also performed to find its proper value. The measured *γ*-ray flux as a function of the horizontal and vertical positions along with the phase are depicted in Fig. 8. They are nicely fitted by Gaussian functions, as expected. The extracted widths are in agreement within 20% with the expected values, according to the accelerator and laser beam parameters and assuming Gaussian distributions. The slight discrepancy is not very significant given the 10% relative error bars but can be also explained by the crude parametrisation used here and some uncertainties related to the actual FPC geometry and the temporal distribution of the laser beam in the presence of thermal effects.

## Additional Information

**How to cite this article**: Chaikovska, I. *et al*. High flux circularly polarized gamma beam factory: coupling a Fabry-Perot optical cavity with an electron storage ring. *Sci. Rep.* **6**, 36569; doi: 10.1038/srep36569 (2016).

**Publisher’s note**: Springer Nature remains neutral with regard to jurisdictional claims in published maps and institutional affiliations.
